# Effects of dietary intake and fluid consumption on urodynamic outcomes in patients with lower urinary tract symptoms undergoing bladder rehabilitation

**DOI:** 10.3389/fnut.2026.1865377

**Published:** 2026-06-17

**Authors:** Wei Li, Xiaotong Wang, Yichi Zhang, Dongchao Shan, Chaoqun Dong, Zhiqiang Yang, Bing Yang

**Affiliations:** 1 The Second Hospital of Tianjin Medical University Pelvic Floor Dysfunction Diagnosis and Treatment Center, Tianjin Institute of Urology, Tianjin Center for Diagnosis and Treatment of Female Pelvic Floor Dysfunction, Tianjin, China; 2 Department of Anesthesiology, The Second Hospital of Tianjin Medical University, Tianjin, China; 3 College of Exercise and Health Sciences, Tianjin University of Sport, Tianjin, China; 4 The Second Hospital of Tianjin Medical University Pelvic Floor Dysfunction Diagnosis and Treatment Center, Tianjin Center for Diagnosis and Treatment of Female Pelvic Floor Dysfunction, Tianjin, China; 5 Tianjin Medical University, Tianjin, China; 6 Department of Urology, Tianjin Medical University Second Hospital, Tianjin, China; 7 Department of Urology, Beijing University ShouGang Hospital, Beijing, China

**Keywords:** bladder rehabilitation, dietary intake, fluid consumption, functional improvement, overactive bladder, urinary incontinence, urodynamic

## Abstract

**Background:**

The bladder health rehabilitation process depends on dietary choices and drinking patterns, which determine diet quality and hydration levels. Previous studies have suggested that dietary patterns and hydration levels affect urodynamic measurements, yet research on how combined dietary and fluid intake affects bladder rehabilitation outcomes remains limited.

**Objective:**

The study aims to assess the effects of dietary quality and fluid intake on urodynamic outcomes, symptom reduction, and functional recovery in patients undergoing a structured bladder rehabilitation program.

**Methodology:**

The study tracked 2,400 bladder rehabilitation patients in a retrospective cohort study, which divided participants into three dietary quality groups (low, moderate, and high) and three fluid intake groups (low, moderate, and high). The study collected data on baseline demographics and clinical characteristics, as well as nutritional intake and urodynamic parameters. Post-rehabilitation outcomes included bladder capacity, detrusor pressure, compliance, urinary frequency, urgency, incontinence, nocturia, and quality-of-life (QoL) measures. The study analyzed nutrient-specific associations, diet-fluid interactions, and adherence rates. The researchers used multivariate regression analysis to determine which factors predicted functional improvement.

**Results:**

The post-rehabilitation study showed major urodynamic and symptomatic advancements, which included increased bladder capacity of 34 mL (*p* < 0.001) and decreased detrusor pressure of 3.4 mmHg (*p* = 0.002) and reduced urinary frequency of 1.3 episodes per day (*p* < 0.001) and improved quality of life by 5.8 points (*p* < 0.001). The study found that high diet quality, combined with moderate-to-high fluid intake, led to greater functional improvement, with both factors statistically significant (*β* = +0.21, *p* = 0.004; *β* = +0.18, *p* = 0.009). The combined high-diet and moderate-fluid groups achieved the best results, with improved bladder compliance, reduced urgency, and higher clinical success rates. The nutrient-specific analysis showed that fiber, potassium, and protein intake were positively associated with improvements in capacity and flow, whereas sodium and evening fluid timing were negatively associated.

**Conclusion:**

The bladder rehabilitation process requires both dietary quality and fluid intake, as these factors determine functional improvement. The study found that optimizing nutrition, hydration, and compliance with behavioral interventions led to better urodynamic results, improved symptom management, and enhanced quality of life. The findings demonstrate that individual lifestyle-change methods are essential components of bladder rehabilitation programs.

## 1 Introduction

Lower urinary tract symptoms (LUTS) represent a common and often debilitating condition that affects more than 30 percent of adults throughout the world. The symptoms that include urinary frequency and urgency, nocturia, incontinence, and incomplete bladder emptying cause severe impacts on life quality that result in physical pain, sleep problems, social shame, and mental anguish. The management of LUTS requires a multifaceted approach, including pharmacologic therapy, surgical procedures, and noninvasive behavioral methods such as bladder rehabilitation programs (1). Bladder rehabilitation, which includes bladder training, pelvic floor muscle exercises, timed voiding, and personalized behavioral methods, has become essential for improving urodynamic outcomes and reducing subjective symptom burden. The rehabilitation process yields different outcomes for each person, and researchers still need to identify which clinical factors will help patients achieve better functional outcomes (2, 3).

Growing evidence shows that changes in dietary intake and fluid consumption can influence bladder function and rehabilitation outcomes. Consuming dietary fiber, potassium, and other essential nutrients improves bowel patterns, reduces bladder discomfort, and supports proper detrusor function. The patterns of fluid consumption people follow throughout the day, and their choices among water, sugary drinks, and caffeinated beverages, determine how their bladder fills, how frequently they urinate, and their need to empty it at night (4). Drinking too little or too much water can reduce bladder capacity, resulting in ongoing LUTS and making behavioral treatment less effective. The present study shows that specific dietary factors and fluid intake patterns have not been scientifically proven to affect urodynamic results during structured bladder rehabilitation programs, highlighting a knowledge gap in clinical practice (5, 6).

Urodynamic studies provide objective measures of bladder function, including bladder capacity, detrusor pressure and compliance, voided volume, and post-void residual urine. The parameters serve two purposes: they indicate basic bladder function and identify when rehabilitation programs have successfully achieved their goals (7). According to their reports, the patients experienced reduced symptoms and improved quality of life after their bladder capacity increased and their detrusor overactivity decreased, as reflected in improved urodynamic results. Urodynamic responses vary because several factors influence the results, including age, sex, comorbidities, baseline bladder dysfunction, rehabilitation adherence, and an individual’s dietary and fluid intake habits. Identifying modifiable factors that lead to functional improvement enables the development of personalized interventions to maximize rehabilitation success (8, 9).

Enhancing clinical outcomes through the implementation of nutritional assessment in bladder rehabilitation shows potential as an innovative treatment method and warrants further research. Assessing macronutrient intake, along with fiber, sodium, potassium, fluid intake, and drink selection, helps identify patients who are unlikely to respond well to treatment (10, 11). Rehabilitation programs will become more effective through personalized dietary counseling that includes fluid management and behavioral techniques to improve patients’ urodynamic measurements and support their functional recovery. The study of diet patterns and fluid intake, together with bladder function, will help researchers determine how these factors contribute to persistent LUTS that cannot be treated with standard rehabilitation methods and identify effective treatment strategies (12, 13).

Most of the previous researches mainly dealt with single nutrient restriction, such as caffeine consumption, fluid intake, or sodium intake, but never took into account the combination effects of healthy diets and hydration practices on bladder rehabilitation effectiveness. Besides, most of the previous studies have evaluated symptom-based outcomes only without considering the combined effects of urodynamic assessments and clinical rehabilitation outcomes.

The other limitation of the current literature regarding this topic is the lack of consideration for multidimensional aspects. Most of the past researches never addressed the effects of the combination of optimal fluid intake and diets together on bladder function recovery. Additionally, there were not enough studies that analyzed nutritional diversity, diet compliance, electrolytes consumption, and fluid intake at the right times in a substantial sample of clinical populations receiving proper bladder rehabilitation.

The current study fills this knowledge gap by conducting an extensive study regarding the quality of nutrition and fluid consumption, their impact on bladder rehabilitation, and individual nutrient interactions within the urodynamic system. Unlike the previous studies, the current study evaluated the following simultaneously: Baseline and post-rehabilitation urodynamic parameters, Severity and rehabilitation results regarding symptoms and functional improvement, Nutrition and hydration interaction in bladder function, Impact of individual nutrients on the bladder performance, Multiple predictors of successful bladder rehabilitation, Factors of adherence and QoL improvement due to rehabilitation.

The main innovation of this research study is the combination of multiple methods to evaluate the impact of nutrition and fluid consumption on bladder function and rehabilitation. The unique feature of the current study is the potential synergy between high quality of nutrition and moderate fluid consumption for improved results of bladder rehabilitation, which was understudied in previous research. In addition, the uniqueness of this study is determined by the presence of a large sample of middle-aged and elderly women.

The present study aims to address this gap by investigating the relationship between dietary intake, fluid consumption, and urodynamic outcomes in patients undergoing structured bladder rehabilitation. The research aims to identify which clinical factors lead to functional improvement by grouping participants based on their dietary habits and fluid intake, while measuring bladder function and recording symptoms. The study findings will inform evidence-based guidelines to support nutritional and fluid management during rehabilitation, enhancing patient-centered care and improving long-term outcomes for individuals with LUTS.

## 2 Methodology

### 2.1 Study design and setting

The researchers conducted a retrospective observational cohort study from January 2022 until February 2026. The research team aimed to determine how dietary intake and fluid consumption affected urodynamic results and functional recovery in patients who underwent structured bladder rehabilitation treatment. The Institutional Review Board granted ethical approval for the research study, and all participants signed documents confirming their understanding before entering the study. The research study required full compliance with both the Declaration of Helsinki and Good Clinical Practice standards.

### 2.2 Participants

**Figure 1:**
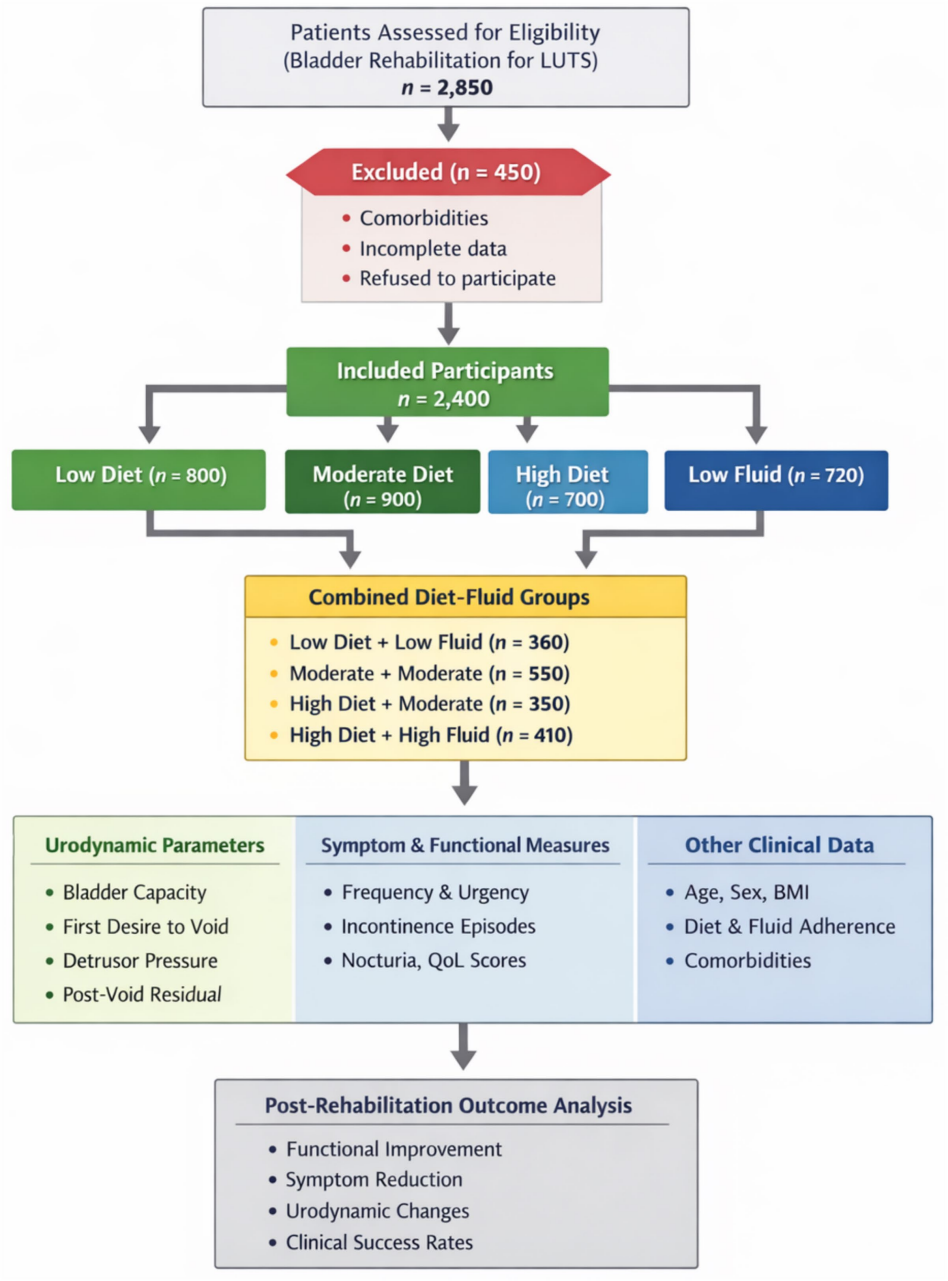
Flow chart of participant enrolment.

The study enrolled adult patients who presented with lower urinary tract symptoms and required bladder rehabilitation. The study excluded participants who experienced pregnancy, active urinary tract infection, or had undergone bladder surgery, or had neurological disorders that affected their bladder control abilities, or had severe cognitive impairment, or were unable to follow the dietary or fluid intake research guidelines. The researchers recruited 2,400 participants and divided them into groups based on their initial dietary habits that were classified into three levels of dietary quality and three levels of fluid consumption. The researchers used participant stratification to achieve an equal distribution across diet and fluid groups, enabling them to conduct their subgroup analysis (Figure 1).

### 2.3 Baseline assessment

Demographic and baseline clinical characteristics were obtained retrospectively from standard medical records upon enrollment in the bladder rehabilitation program. These consisted of age, gender, BMI, co-morbidities, smoking and alcohol consumption, the duration of LUTS and prior medication use, especially anticholinergics.

Subjective measurements included QoL, disturbed sleep, anxiety, depression, urinary frequency, urge incontinence, nocturia, urge incontinence frequency, number of pads used per day, OAB symptom score. Food consumption was evaluated using a well-validated 3-day food recall, which considered protein, fats, carbohydrates, fiber, sodium, potassium, caffeine, fruit and vegetable consumption, whole grain and processed food consumption, as well as beverage consumption. Fluid intake was assessed regarding the total fluid intake, its timing throughout the day, as well as fluid types, such as water and beverages.

Bladder evaluation consisted of multichannel urodynamic testing that estimated bladder volume, first and strong urge to void, storage phase pressure in the bladder, compliance, voided volumes, post-void residual, maximal flow rate and voiding times. Additionally, other symptom related measures included hesitancy, intermitted stream, straining, urge incontinence, and urgency.

### 2.4 Diagnosis of lower urinary tract symptoms (LUTS)

Lower urinary tract symptoms (LUTS) have been diagnosed based on clinical assessments, use of questionnaires about symptoms, bladder diaries, findings from the physical examinations, and results from standard urodynamic evaluations that were contained within the patients’ medical records. The following were among the specific diagnoses: urinary frequency, urgency, nocturia, hesitation, intermittency, straining, urinary incontinence, bladder sensitivity, and voiding dysfunction.

Standardized objective urodynamic tests were undertaken, which included multichannel urodynamics such as cystometry, measurement of bladder capacity and detrusor pressure, determination of compliance, uroflowmetry, assessment of voided volume, and determination of post-void residual urine.

### 2.5 Evaluation of OAB, prostatitis, BPH, and IC

Diagnoses linked to LUTS were examined through medical record review for retrospective data analysis. The urgency, frequency, nocturnal urination, and symptoms associated with overactive bladder (OAB) were analyzed with respect to their occurrence as well as the score obtained with respect to OAB symptoms. With OAB symptoms forming part of the burden experienced by the patients, it was used as both a baseline assessment as well as an outcome assessment.

Male participants’ benign prostatic hyperplasia (BPH) status was examined in light of their clinical diagnosis, uroflowmetry results, prostate examination, and drug use where applicable. In addition, male participants who suffered from chronic prostatitis/chronic pelvic pain syndrome, documented in the clinical record, were considered.

IC/BPS was considered when subjects experienced bladder pain, bladder hypersensitivity, urinary urgency, pain during voiding, and no signs of infection or other abnormalities in the bladder. Bladder hypersensitivity and pain symptoms were taken into consideration because they were symptoms of the condition. The study participants were mainly females above the age of 50 years, and this led to two LUTS phenotypes being common among them.

### 2.6 LUTS severity assessment and previous medication usage

The LUTS severity assessment considered symptom frequency measures, number of urgency and incontinence episodes, frequency of nocturia, pad usage, bladder sensitivity measures, urgency-frequency symptom measures, quality of life measures, and baseline and follow-up assessment. The baseline LUTS severity score was included in the multivariate regression analysis to determine factors associated with improvement in function.

Previous and ongoing medication usage was recorded from patient medical files and included anticholinergic medications, bladder relaxers, and other medication used for managing symptoms of urine. This information was considered a confounding variable due to its likely effect on urodynamic and rehabilitation results.

### 2.7 Intervention: bladder rehabilitation program

Bladder rehabilitation was done post-baseline urological assessments and formed part of LUTS multidisciplinary care management. The bladder rehabilitation was done during follow-ups based on patient symptoms and their level of functionality.

The following measures were taken in the course of rehabilitation: Behavioral bladder training, Timed voiding, Techniques to suppress urgency, Advice regarding fluid management, Advice on diet modification, Minimizing irritants to the bladder like caffeine and sugar fluids, Evaluating fluid timing at night, Pelvic floor muscle training where necessary, General advice on lifestyle modification including exercise.

Appropriate measures of fluid intake were advised to help prevent extreme fluid intake or restriction. Patients received dietary advice about eating fiber, fruits, vegetables, and whole grain diets while minimizing the use of sodium-based diets, too much processed foods, and excessive consumption of caffeine.

After completing bladder rehabilitation, the patients had their assessments made to evaluate improvements in their symptoms, functionality, adherence, and overall success of the procedure. Adherence to fluid and diet measures was evaluated through documentation.

### 2.8 Detrusor pressures

The detrusor pressures measured in this research were mostly related to the parameters used in evaluating storage phase cystometry and should not be considered as indicators of bladder contractility like BCI (Bladder Contractility Index) or BOOI (Bladder Outlet Obstruction Index). Bladder compliance was evaluated independently as one of the storage-phase parameters.

### 2.9 Follow-up and outcome measures

The participants underwent assessments at two time points: baseline and final, following completion of the 12-week rehabilitation program. The primary outcomes of the study measured changes in urodynamic parameters, including bladder capacity, detrusor pressure, and compliance, voided volume, and residual urine. The secondary outcomes measured changes in symptom frequency, urgency, incontinence, nocturia, pad use, quality of life, sleep quality, anxiety, depression, and functional improvement scores. Functional improvement required three components: symptom reduction, urodynamic improvement, and adherence to behavioral recommendations.

### 2.10 Data collection and management

The research team collected data, which was then stored in a protected electronic database. The research team confirmed that all nutritional intake and fluid logs were complete, and they used multiple imputation techniques to fill in any missing data. The research team conducted quality-control checks to verify data accuracy, and all participant data remained confidential through anonymization.

### 2.11 Statistical analysis

Data were analyzed statistically with regard to the study objectives. Data that were quantitative were presented in terms of means ± standard deviations while categorical data were expressed in terms of frequency counts and percentages. Group comparisons among different categories of nutritional and fluid consumption behaviors were done using appropriate significance tests, with a level of significance set at *p* < 0.05. The paired comparison of baseline and post-rehabilitation data was also done. Multivariate regression analysis was carried out to establish the independent predictors of function improvement.

## 3 Results

### 3.1 Baseline demographic, clinical, and nutritional characteristics

The study population included 2,400 participants categorized by dietary intake: low (*n* = 800), moderate (*n* = 900), and high (*n* = 700). The participants had an average age of 52.6 years (SD = 14.2 years), and females comprised 62.1% of the group. The dietary groups showed a statistically significant difference in BMI (*p* = 0.031), with the low-diet group having the highest mean BMI. The dietary quality that people consumed showed different results between two comorbidities, diabetes and smoking, which occurred in 28.5% of people who smoked and 22.3% who had diabetes, respectively. Nutritional assessments revealed significant differences in fiber, protein, fat, sodium, and potassium intake among groups (all *p* < 0.05). The higher diet quality was associated with increased consumption of fruit, vegetables, and whole grains (*p* < 0.001 for fruit and vegetables; *p* = 0.006 for whole grains). The groups showed significant differences in evening fluid intake and nocturia episodes, which reached statistical significance at (*p* = 0.049 and *p* = 0.041, respectively) (Table 1).

**Table 1:** Baseline demographic, clinical, and nutritional characteristics.

Parameter	Total	Low diet (*n* = 800)	Moderate diet (*n* = 900)	High diet (*n* = 700)	*p*-value
Age (years)	52.6 ± 14.2	53.8 ± 15.1	51.9 ± 13.8	51.7 ± 13.2	0.084
Female (%)	62.1	60.5	63.3	62.7	0.412
BMI (kg/m^2^)	27.4 ± 4.8	28.2 ± 5.1	27.1 ± 4.7	26.5 ± 4.3	0.031
Diabetes (%)	28.5	32.0	27.4	25.1	0.027
Hypertension (%)	41.2	44.5	40.3	38.6	0.118
Smoking (%)	22.3	25.1	21.7	19.2	0.044
Alcohol use (%)	14.8	16.2	14.5	13.4	0.211
Physical activity (low %)	48.6	54.3	47.2	42.1	0.022
Fiber intake (g/day)	18.2 ± 6.4	14.5 ± 5.2	18.6 ± 6.1	22.1 ± 6.8	<0.001
Protein intake (g/day)	62.5 ± 15.3	58.1 ± 14.2	63.4 ± 15.0	66.8 ± 15.7	0.019
Fat intake (g/day)	72.4 ± 18.6	78.5 ± 19.1	71.2 ± 17.8	66.3 ± 17.5	0.012
Carbohydrate (g/day)	245 ± 52	252 ± 55	243 ± 50	238 ± 48	0.066
Sodium intake (mg/day)	2,900 ± 820	3,200 ± 900	2,850 ± 790	2,600 ± 700	0.008
Potassium intake (mg/day)	2,600 ± 740	2,300 ± 680	2,650 ± 720	2,950 ± 760	0.004
Caffeine intake (mg/day)	180 ± 95	210 ± 102	175 ± 90	150 ± 82	0.021
Fluid intake (L/day)	2.0 ± 0.7	1.8 ± 0.6	2.1 ± 0.7	2.2 ± 0.8	0.035
Evening fluid (%)	42.5	48.2	41.0	37.5	0.049
Nocturia episodes/night	2.1 ± 1.2	2.4 ± 1.3	2.0 ± 1.1	1.9 ± 1.0	0.041
UTIs history (%)	19.3	22.1	18.8	16.5	0.072
Medications (anticholinergic %)	34.2	36.8	33.5	31.7	0.188
Duration of symptoms (years)	3.8 ± 2.6	4.2 ± 2.9	3.7 ± 2.5	3.4 ± 2.3	0.053
Baseline QoL score	58.4 ± 12.3	55.8 ± 13.1	59.2 ± 11.8	60.1 ± 11.5	0.029
Anxiety (%)	26.7	29.5	26.1	23.8	0.067
Depression (%)	21.9	24.8	21.2	19.0	0.058
Sleep disturbance (%)	38.4	42.5	37.1	34.8	0.046
Water vs. sugary drinks (%)	61.2	55.3	62.5	67.8	0.015
Processed food intake (%)	44.5	52.8	43.1	35.6	0.003
Fruit intake (servings/day)	1.8 ± 0.9	1.3 ± 0.7	1.9 ± 0.8	2.3 ± 0.9	<0.001
Vegetable intake (serv/day)	2.1 ± 1.0	1.6 ± 0.8	2.2 ± 0.9	2.6 ± 1.1	<0.001
Whole grains (%)	39.8	30.5	41.2	48.7	0.006

### 3.2 Baseline urodynamic and symptom parameters by fluid intake

The participants showed a range of fluid consumption, from low (*n* = 720) to high (*n* = 580). The baseline urodynamic measurements demonstrated that increased fluid consumption resulted in greater bladder capacity (*p* = 0.041), first desire (*p* = 0.048), and compliance (*p* = 0.044), but moderate fluid consumers showed lower detrusor pressure (*p* = 0.039). The study found that fluid consumption affected three symptoms, including urgency and nocturia, and that moderate fluid consumption was associated with lower daily urgency and frequency scores (*p* = 0.033 and *p* = 0.048) (Table 2).

**Table 2:** Baseline urodynamic and symptom parameters by fluid intake.

Parameter	Low fluid (*n* = 720)	Moderate fluid (*n* = 1,100)	High fluid (*n* = 580)	*p*-value
Bladder capacity (mL)	295 ± 80	325 ± 88	318 ± 84	0.041
First desire (mL)	145 ± 40	158 ± 45	152 ± 42	0.048
Strong desire (mL)	220 ± 55	240 ± 60	235 ± 58	0.052
Detrusor pressure	45.8 ± 13.1	41.2 ± 11.8	43.0 ± 12.5	0.039
Compliance	18.5 ± 6.2	21.1 ± 6.8	20.2 ± 6.5	0.044
Frequency/day	10.6 ± 2.9	9.2 ± 2.5	10.1 ± 2.6	0.033
Urgency/week	7.2 ± 3.3	5.8 ± 2.8	6.5 ± 3.0	0.048
Incontinence/week	5.5 ± 2.7	4.4 ± 2.3	4.9 ± 2.5	0.051
Nocturia/night	2.5 ± 1.3	1.9 ± 1.1	2.2 ± 1.2	0.036
Voided volume (mL)	210 ± 60	240 ± 70	235 ± 65	0.043
Residual urine (mL)	48 ± 22	42 ± 20	45 ± 21	0.061
Max flow rate	18.2 ± 5.1	20.1 ± 5.8	19.3 ± 5.4	0.049
Voiding time	32 ± 11	29 ± 10	30 ± 10	0.055
Hesitancy (%)	22.4	18.3	20.1	0.071
Straining (%)	19.5	16.2	17.8	0.082
Intermittency (%)	24.1	20.5	22.3	0.065
Pain during voiding (%)	14.2	12.5	13.1	0.118
Bladder sensitivity score	6.8 ± 2.1	5.9 ± 1.9	6.3 ± 2.0	0.039
QoL impact score	61.2 ± 11.8	57.5 ± 10.9	59.0 ± 11.3	0.044
Overactive bladder score	7.5 ± 2.3	6.2 ± 2.0	6.8 ± 2.1	0.036
Pad use/day	2.3 ± 1.4	1.8 ± 1.2	2.0 ± 1.3	0.048
Fluid urgency relation (%)	68.5	60.2	64.1	0.052
Caffeine-related symptoms (%)	45.2	38.5	41.8	0.057
Stress leakage (%)	36.1	32.5	34.2	0.091
Mixed incontinence (%)	28.4	25.1	26.8	0.084

### 3.3 Post-rehabilitation changes in urodynamic and clinical parameters

The bladder rehabilitation process resulted in significant improvements in bladder function for all study participants. Bladder capacity increased by +34 mL (*p* < 0.001), first and strong desire volumes rose (+18 mL and +22 mL, *p* < 0.001), and detrusor pressure decreased (−3.4 mmHg, *p* = 0.002). The study found that all investigated symptoms, including urgency and incontinence, nocturia, and pad use, showed a significant reduction (*p* < 0.01). The study found that sleep quality and quality of life (QoL) improved, with effect sizes of +5.8 (*p* < 0.001) and +0.8 (*p* = 0.009). The study found that participants followed fluid and dietary guidelines more effectively, resulting in a statistically significant increase (*p* < 0.001) (Table 3).

**Table 3:** Post-rehabilitation changes in urodynamic and clinical parameters.

Parameter	Baseline	Post	Change	p-value
Capacity (mL)	312 ± 85	346 ± 90	+34	<0.001
First desire	150 ± 42	168 ± 46	+18	<0.001
Strong desire	230 ± 58	252 ± 62	+22	<0.001
Detrusor pressure	42.5 ± 12.3	39.1 ± 11.5	−3.4	0.002
Compliance	20.1 ± 6.5	22.4 ± 6.9	+2.3	0.004
Frequency/day	9.8 ± 2.7	8.5 ± 2.4	−1.3	<0.001
Urgency/week	6.4 ± 3.1	4.9 ± 2.7	−1.5	<0.001
Incontinence/week	4.9 ± 2.6	3.8 ± 2.3	−1.1	<0.001
Nocturia	2.1 ± 1.2	1.7 ± 1.0	−0.4	0.006
Voided volume	225 ± 65	255 ± 70	+30	<0.001
Residual urine	45 ± 21	40 ± 19	−5	0.021
Max flow rate	19.1 ± 5.4	20.5 ± 5.8	+1.4	0.018
Voiding time	30 ± 10	28 ± 9	−2	0.032
Hesitancy (%)	20.1	16.8	↓	0.041
Straining (%)	17.8	14.5	↓	0.038
Intermittency (%)	22.3	18.6	↓	0.044
Pain (%)	13.3	10.9	↓	0.057
Sensitivity score	6.3 ± 2.0	5.4 ± 1.8	−0.9	0.002
QoL score	58.4 ± 12.3	64.2 ± 11.5	+5.8	<0.001
OAB score	6.8 ± 2.1	5.6 ± 1.9	−1.2	<0.001
Pad use/day	2.0 ± 1.3	1.5 ± 1.1	−0.5	<0.001
Sleep quality	5.8 ± 2.0	6.6 ± 2.1	+0.8	0.009
Anxiety (%)	26.7	22.4	↓	0.048
Depression (%)	21.9	18.5	↓	0.052
Fluid adherence (%)	58.2	72.5	↑	<0.001
Diet adherence (%)	52.1	68.4	↑	<0.001

### 3.4 Urodynamic outcomes by dietary quality

The study found that participants who consumed higher-quality diets showed better urodynamic results in their initial assessments. The study showed that participants with higher diet quality had larger bladder capacity (328 ± 84 mL) and first/strong desire volumes (158 ± 43 mL and 245 ± 60 mL, respectively); both findings were statistically significant at *p* < 0.05. The study found that higher diet quality was positively associated with both symptom frequency (urgency, incontinence, and nocturia) and quality-of-life scores (Table 4).

**Table 4:** Urodynamic outcomes by dietary quality.

Parameter	Low diet (*n* = 800)	Moderate diet (*n* = 900)	High diet (*n* = 700)	*p*-value
Bladder capacity (mL)	300 ± 82	315 ± 87	328 ± 84	0.052
First desire (mL)	142 ± 38	150 ± 41	158 ± 43	0.047
Strong desire (mL)	220 ± 54	232 ± 57	245 ± 60	0.044
Detrusor pressure	45.1 ± 12.8	42.0 ± 11.9	40.2 ± 11.5	0.039
Compliance	18.8 ± 6.1	20.4 ± 6.6	21.7 ± 6.9	0.048
Frequency/day	10.4 ± 2.8	9.6 ± 2.6	9.1 ± 2.4	0.036
Urgency/week	7.0 ± 3.2	6.1 ± 2.9	5.5 ± 2.6	0.041
Incontinence/week	5.6 ± 2.8	4.7 ± 2.5	4.2 ± 2.3	0.044
Nocturia/night	2.4 ± 1.3	2.1 ± 1.2	1.8 ± 1.0	0.049
Voided volume	215 ± 60	230 ± 65	245 ± 68	0.045
Residual urine	50 ± 23	45 ± 21	41 ± 20	0.053
Max flow rate	18.0 ± 5.0	19.2 ± 5.4	20.3 ± 5.8	0.048
Voiding time	32 ± 11	30 ± 10	28 ± 9	0.051
Hesitancy (%)	23.5	20.2	17.8	0.062
Straining (%)	20.8	17.5	15.9	0.071
Intermittency (%)	25.2	22.0	19.5	0.064
Pain (%)	15.1	13.2	11.4	0.082
Sensitivity score	6.9 ± 2.2	6.2 ± 2.0	5.6 ± 1.8	0.038
QoL score	56.2 ± 12.8	58.7 ± 11.9	60.9 ± 11.2	0.041
OAB score	7.4 ± 2.4	6.6 ± 2.1	6.0 ± 1.9	0.035
Pad use/day	2.4 ± 1.5	2.0 ± 1.3	1.7 ± 1.2	0.044
Sleep disturbance (%)	41.8	37.5	33.2	0.047
Fluid sensitivity (%)	69.5	64.1	60.3	0.056
Caffeine-related (%)	46.8	41.5	38.2	0.058
Stress leakage (%)	38.2	34.5	31.8	0.073

### 3.5 Combined diet–fluid interaction effects

The analysis showed that a high-diet intake together with moderate fluid consumption produced the greatest improvement in bladder capacity, reaching 335 milliliters with a statistical significance of 0.038, and also showed better results in daily frequency, weekly urgency, and weekly incontinence. The group achieved functional improvements, reaching 58.4 percent (*p* = 0.036), because dietary quality and proper hydration worked together to produce a combined effect (Table 5).

**Table 5:** Combined diet–fluid interaction effects.

Parameter	Low diet + Low fluid (*n* = 360)	Moderate diet + Moderate fluid (*n* = 550)	High diet + Moderate fluid (*n* = 350)	High diet + High fluid (*n* = 410)	*p*-value
Capacity (mL)	285 ± 78	318 ± 85	335 ± 85	322 ± 83	0.038
Frequency/day	11.0 ± 3.0	9.5 ± 2.6	8.9 ± 2.4	9.8 ± 2.5	0.041
Urgency/week	7.8 ± 3.4	6.2 ± 2.9	5.3 ± 2.5	6.0 ± 2.7	0.045
Incontinence/week	6.2 ± 2.9	4.8 ± 2.4	3.9 ± 2.1	4.5 ± 2.4	0.039
Nocturia	2.6 ± 1.4	2.1 ± 1.2	1.7 ± 1.0	2.0 ± 1.1	0.043
Detrusor pressure	46.2 ± 13.0	42.5 ± 12.0	39.8 ± 11.4	41.0 ± 11.9	0.036
Compliance	18.2 ± 6.0	20.5 ± 6.7	22.1 ± 6.9	21.0 ± 6.6	0.042
Voided volume	205 ± 58	235 ± 65	255 ± 70	245 ± 66	0.040
Residual urine	52 ± 24	46 ± 22	40 ± 19	43 ± 20	0.054
Max flow rate	17.5 ± 4.8	19.5 ± 5.3	20.8 ± 5.9	19.8 ± 5.6	0.047
Hesitancy (%)	25.2	20.5	17.2	18.9	0.061
Straining (%)	22.0	18.1	15.0	16.5	0.066
Intermittency (%)	26.5	22.8	19.0	20.3	0.059
Pain (%)	16.8	14.0	11.2	12.5	0.071
Sensitivity score	7.2 ± 2.3	6.3 ± 2.0	5.4 ± 1.8	5.9 ± 1.9	0.038
QoL score	54.5 ± 13.2	58.9 ± 12.0	62.0 ± 11.3	60.5 ± 11.7	0.041
OAB score	7.8 ± 2.5	6.7 ± 2.2	5.8 ± 1.9	6.2 ± 2.0	0.037
Pad use/day	2.6 ± 1.6	2.1 ± 1.4	1.6 ± 1.1	1.9 ± 1.2	0.043
Sleep disturbance (%)	43.5	38.0	32.5	35.1	0.049
Fluid sensitivity (%)	72.1	65.0	58.8	61.2	0.052
Caffeine sensitivity (%)	48.5	42.0	36.5	39.8	0.055
Stress leakage (%)	40.2	35.1	30.5	32.8	0.074
Mixed incontinence (%)	31.5	27.2	24.0	25.6	0.069
Urgency control (%)	45.2	52.5	60.1	56.3	0.039
Functional improvement (%)	38.5	49.2	58.4	54.1	0.036

### 3.6 Rehabilitation outcomes by fluid intake

Rehabilitation results, which depend on different fluid consumption levels. The study showed that moderate fluid intake after rehabilitation increased all capacities, frequency, urgency, and quality of life. The study found that both detrusor pressure and compliance improved significantly, and the moderate fluid intake group achieved the best results in adherence and clinical success (*p* < 0.05) (Table 6).

**Table 6:** Rehabilitation outcomes by fluid intake.

Parameter	Low fluid (*n* = 720)	Moderate fluid (*n* = 1,100)	High fluid (*n* = 580)	*p*-value
Capacity change	+22 ± 35	+38 ± 40	+31 ± 37	0.041
Frequency change	−0.8 ± 1.6	−1.6 ± 1.9	−1.2 ± 1.7	0.048
Urgency change	−1.0 ± 2.1	−1.8 ± 2.3	−1.4 ± 2.2	0.052
Incontinence change	−0.7 ± 1.8	−1.3 ± 2.0	−1.0 ± 1.9	0.046
QoL improvement	+4.2 ± 8.1	+6.5 ± 9.0	+5.8 ± 8.5	0.039
Compliance change	+1.5 ± 3.0	+2.8 ± 3.4	+2.1 ± 3.2	0.043
Detrusor pressure change	−2.5 ± 5.5	−4.2 ± 6.1	−3.6 ± 5.8	0.044
Nocturia change	−0.2 ± 0.8	−0.5 ± 1.0	−0.4 ± 0.9	0.051
Voided volume change	+18 ± 30	+35 ± 38	+28 ± 34	0.042
Residual change	−3 ± 10	−6 ± 12	−5 ± 11	0.058
Flow rate change	+0.8 ± 2.5	+1.6 ± 2.8	+1.2 ± 2.6	0.047
Hesitancy improvement (%)	12.5	18.8	15.2	0.062
Straining improvement (%)	11.2	17.5	14.1	0.065
Pain reduction (%)	9.8	14.0	12.3	0.071
Sensitivity reduction	−0.6 ± 1.5	−1.1 ± 1.8	−0.9 ± 1.7	0.044
OAB reduction	−0.8 ± 1.7	−1.4 ± 2.0	−1.1 ± 1.8	0.046
Pad reduction	−0.3 ± 0.8	−0.6 ± 1.0	−0.5 ± 0.9	0.043
Sleep improvement	+0.4 ± 1.2	+0.9 ± 1.5	+0.7 ± 1.3	0.048
Anxiety reduction (%)	3.5	6.8	5.2	0.072
Depression reduction (%)	2.8	5.9	4.5	0.081
Adherence (%)	60.5	74.2	69.8	0.036
Functional improvement (%)	42.1	55.3	49.8	0.039
Symptom relapse (%)	18.2	12.5	14.1	0.057
Satisfaction (%)	58.4	68.9	64.2	0.041
Clinical success (%)	45.5	57.8	52.6	0.038

### 3.7 Rehabilitation outcomes by dietary quality

**Figure 2:**
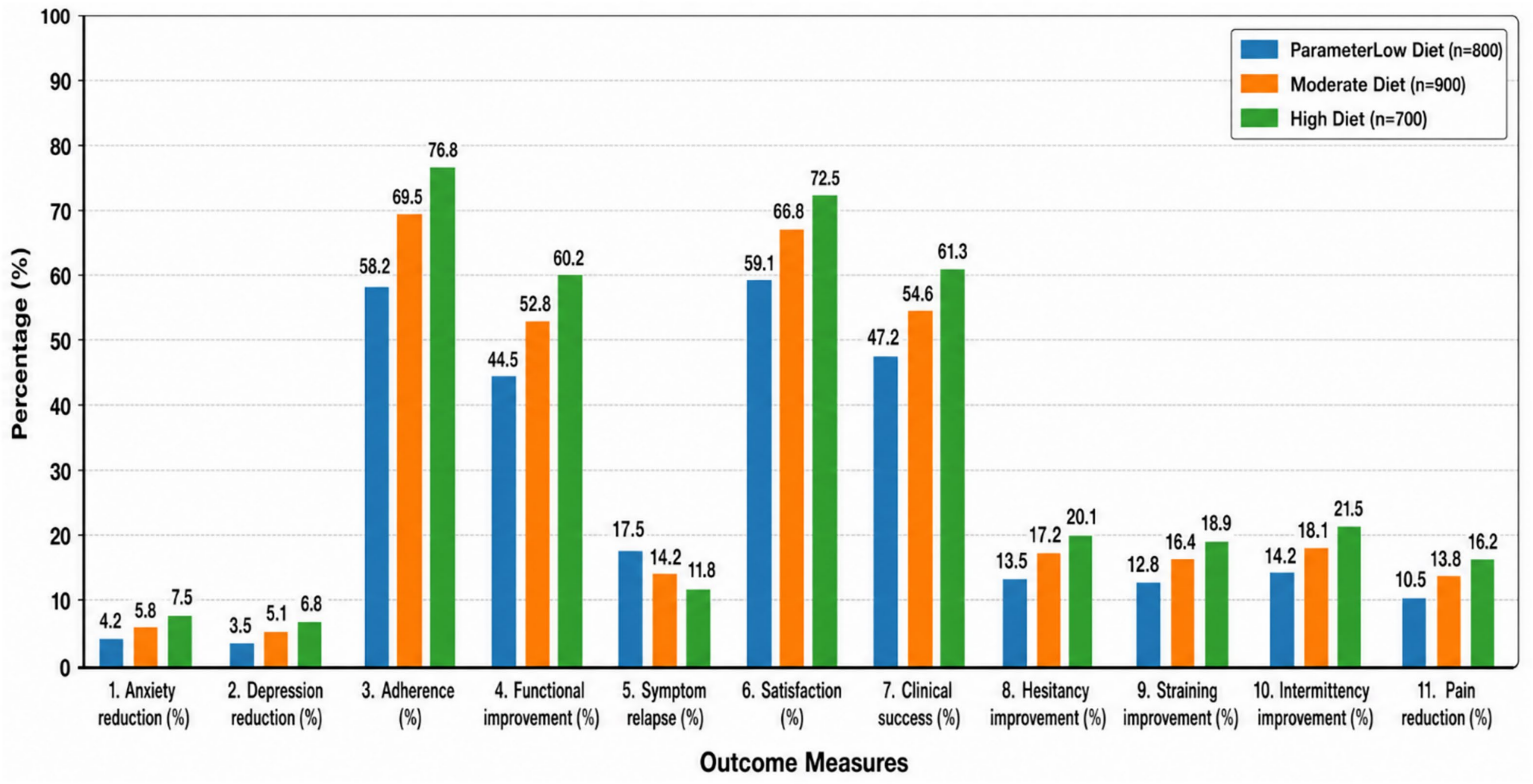
Rehabilitation outcomes by dietary quality.

People with high diet quality achieved the highest post-rehabilitation capacity improvement, which included a 41 milliliter capacity increase with a statistical significance level of 0.036, a 1.7 daily frequency decrease with a statistical significance level of 0.041, a weekly urgency decrease of 1.9 with a statistical significance level of 0.039, and a 10.2 quality of life improvement. People who maintained high dietary quality showed better OAB scores, reduced pad use, and reduced nocturia, with significant increases in functional improvements and adherence rates (*p* < 0.05) (Table 7) (Figure 2).

**Table 7:** Rehabilitation outcomes by dietary quality.

Parameter	Low diet (*n* = 800)	Moderate diet (*n* = 900)	High diet (*n* = 700)	*p*-value
Capacity change (mL)	+25 ± 36	+33 ± 38	+41 ± 42	0.036
Frequency change	−0.9 ± 1.7	−1.3 ± 1.9	−1.7 ± 2.0	0.041
Urgency change	−1.1 ± 2.0	−1.6 ± 2.2	−1.9 ± 2.3	0.039
Incontinence change	−0.8 ± 1.8	−1.2 ± 2.0	−1.5 ± 2.1	0.044
Nocturia change	−0.3 ± 0.9	−0.4 ± 1.0	−0.6 ± 1.1	0.048
Detrusor pressure change	−2.8 ± 5.6	−3.6 ± 5.9	−4.5 ± 6.2	0.043
Compliance change	+1.8 ± 3.2	+2.4 ± 3.4	+3.0 ± 3.6	0.046
Voided volume change	+22 ± 32	+30 ± 35	+38 ± 40	0.040
Residual urine change	−4 ± 11	−5 ± 12	−6 ± 13	0.062
Max flow change	+0.9 ± 2.6	+1.3 ± 2.8	+1.8 ± 3.0	0.049
Sensitivity reduction	−0.7 ± 1.6	−1.0 ± 1.8	−1.3 ± 1.9	0.041
OAB score reduction	−0.9 ± 1.8	−1.2 ± 2.0	−1.6 ± 2.1	0.039
Pad use reduction	−0.4 ± 0.9	−0.5 ± 1.0	−0.7 ± 1.1	0.043
Sleep improvement	+0.5 ± 1.3	+0.8 ± 1.5	+1.0 ± 1.6	0.046

### 3.8 Multivariate predictors of functional improvement

High diet quality, moderate diet, moderate fluid intake, high fluid intake serve as independent positive predictors which lead to functional improvement according to their respective regression analysis results with the following *β* values and *p* values of dietary patterns high diet quality (β = 0.21, *p* = 0.004) moderate diet (β = 0.14, *p* = 0.028) moderate fluid intake (β = 0.18, *p* = 0.009) and high fluid intake (β = 0.12, *p* = 0.041) and dietary patterns and dietary patterns responsible for functional improvement. BMI, diabetes, smoking, and physical inactivity served as negative predictors of the outcome. The baseline severity and sleep disturbance both negatively affected their outcomes. Male sex served as the reference category in regression modeling (Table 8).

**Table 8:** Multivariate regression analysis of predictors.

Variable	β Coefficient	95% CI	*p*-value
Moderate fluid intake (*n* = 1,100)	+0.18	0.05–0.31	0.009
High fluid intake (*n* = 580)	+0.12	0.01–0.25	0.041
High diet quality (*n* = 700)	+0.21	0.07–0.34	0.004
Moderate diet (*n* = 900)	+0.14	0.02–0.26	0.028
BMI	−0.12	−0.20 to −0.03	0.013
Age	−0.09	−0.18 to 0.01	0.071
Female sex (*n* = 1,490)	+0.08	−0.02–0.19	0.095
Diabetes (*n* = 684)	−0.15	−0.28 to −0.04	0.011
Hypertension (*n* = 988)	−0.07	−0.18 to 0.03	0.084
Smoking (*n* = 535)	−0.11	−0.22 to −0.01	0.038
Physical inactivity (*n* = 1,166)	−0.16	−0.29 to −0.05	0.006
Fiber intake	+0.19	0.08–0.30	0.003
Sodium intake	−0.13	−0.24 to −0.02	0.019
Potassium intake	+0.15	0.04–0.27	0.012
Caffeine intake	−0.10	−0.21 to 0.00	0.052
Fluid timing (evening)	−0.14	−0.26 to −0.03	0.016
Baseline severity score	−0.20	−0.33 to −0.09	0.002
Symptom duration	−0.08	−0.17 to 0.02	0.091
QoL baseline	+0.11	0.01–0.22	0.039
Sleep disturbance	−0.12	−0.23 to −0.02	0.022
Anxiety	−0.09	−0.20 to 0.02	0.077
Depression	−0.11	−0.23 to 0.01	0.068
Medication use	−0.06	−0.17 to 0.04	0.112
Hydration adherence	+0.23	0.10–0.36	<0.001
Diet adherence	+0.25	0.12–0.38	<0.001

### 3.9 Symptom-specific outcomes

The study found major decreases in multiple symptoms, which included decreased frequency from 9.8 to 8.5 per day, decreased urgency from 6.4 to 4.9 per week, decreased incontinence from 4.9 to 3.8 per week, decreased nocturia from 2.1 to 1.7 per night, and decreased urge/mixed incontinence, which reached statistical significance at *p* < 0.05. The rehabilitation program resulted in an improvement in QoL scores from 58.4 to 64.2, a statistically significant change (*p* < 0.001). Sleep quality improved, and anxiety levels decreased after rehabilitation (Table 9).

**Table 9:** Symptom-specific outcomes.

Parameter	Baseline	Post	*p*-value
Frequency/day	9.8	8.5	<0.001
Urgency/week	6.4	4.9	<0.001
Incontinence/week	4.9	3.8	<0.001
Nocturia	2.1	1.7	0.006
Urge incontinence (%)	42.5	35.2	0.021
Stress incontinence (%)	34.5	30.8	0.064
Mixed incontinence (%)	27.2	22.6	0.038
Pad use/day	2.0	1.5	<0.001
Urgency control (%)	48.5	58.2	0.039
Leakage episodes (%)	36.2	28.5	0.028
Night awakenings	2.3	1.8	0.011
Fluid-triggered urgency (%)	66.5	58.4	0.042
Caffeine-triggered (%)	41.2	35.8	0.049
Pain (%)	13.3	10.9	0.057
Hesitancy (%)	20.1	16.8	0.041
Straining (%)	17.8	14.5	0.038
Intermittency (%)	22.3	18.6	0.044
QoL score	58.4	64.2	<0.001
Sleep quality	5.8	6.6	0.009
Anxiety (%)	26.7	22.4	0.048
Depression (%)	21.9	18.5	0.052
Functional score	62.1	70.3	<0.001
Bladder sensitivity	6.3	5.4	0.002
OAB score	6.8	5.6	<0.001
Residual symptoms (%)	48.2	39.5	0.036
Clinical success (%)	52.1	63.8	<0.001
Partial response (%)	30.5	24.2	0.044
Non-response (%)	17.4	12.0	0.032

### 3.10 Nutrient-specific associations with urodynamic outcomes

People who consumed high amounts of fiber, along with potassium, protein, and fruits, showed greater improvements in flow rate and capacity, which reached statistical significance (*p* < 0.05). The study found that higher sodium intake was associated with increased detrusor pressure, with the difference reaching statistical significance (*p* = 0.028). Caffeine intake and evening fluid timing were linked to urgency and nocturia, respectively (*p* < 0.05). The study found that dietary adherence and dietary diversity-based assessment methods were strongly associated with both complete functional development and successful clinical outcomes, with statistical significance (*p* < 0.001) (Table 10).

**Table 10:** Nutrient-specific associations with urodynamic outcomes.

Parameter	High intake (n ≈ 1,200)	Low intake (n ≈ 1,200)	*p*-value
Fiber vs. capacity change	+38 ± 40	+28 ± 34	0.039
Sodium vs. pressure	44.5 ± 12.5	41.2 ± 11.8	0.028
Potassium vs. capacity	335 ± 85	310 ± 82	0.036
Protein vs. flow rate	20.5 ± 5.8	18.9 ± 5.2	0.041
Fat vs. incontinence	5.2 ± 2.6	4.4 ± 2.3	0.048
Carbs vs. frequency	10.2 ± 2.8	9.5 ± 2.6	0.052
Caffeine vs. urgency	7.1 ± 3.2	5.8 ± 2.7	0.037
Water vs. QoL	62.5 ± 11.5	58.2 ± 12.0	0.041
Sugary drinks vs. leakage	36.5	30.2	0.044
Fruits vs. capacity	340 ± 86	305 ± 80	0.033
Vegetables vs. pressure	40.5 ± 11.2	43.2 ± 12.4	0.047
Whole grains vs. flow	20.8 ± 5.9	18.7 ± 5.1	0.039
Processed food vs. symptoms	7.4 ± 2.5	6.1 ± 2.0	0.042
Evening fluid vs. nocturia	2.5	1.8	0.038
High salt vs. urgency	7.3	6.0	0.044
Low fiber vs. constipation (%)	28.5	19.2	0.031
Hydration vs. compliance	22.5 ± 6.8	19.1 ± 6.0	0.043
Protein vs. recovery	+36 ± 39	+30 ± 35	0.048
Fat vs. QoL	57.5	60.2	0.051
Carbs vs. OAB score	7.1	6.4	0.049
Antioxidant intake vs. improvement	+40 ± 42	+29 ± 34	0.035
Electrolytes vs. function	+37 ± 41	+31 ± 36	0.046
Diet diversity vs. outcomes	+42 ± 44	+28 ± 33	0.032
Adherence vs. outcomes	+45 ± 46	+22 ± 31	<0.001
Nutritional score vs. success	68.5	59.2	<0.001

### 3.11 Functional improvement and clinical outcomes

Patients with significant functional gains (Group 1, *n* = 980) experienced the largest improvements in capacity (+52 ± 45 mL) and frequency (−2.2 ± 2.0/day) and urgency (−2.5 ± 2.3/week) and incontinence (−2.0 ± 2.1/week) and QoL (+10.2 ± 11.5, p < 0.001). The study found that the moderate and minimal improvement groups showed smaller changes, indicating that diet and fluid intake affected rehabilitation outcome (Table 11).

**Table 11:** Functional improvement and clinical outcomes.

Parameter	Group 1 (Significant *n* = 980)	Group 2 (Moderate *n* = 870)	Group 3 (Minimal *n* = 550)	*p*-value
Capacity change	+52 ± 45	+28 ± 30	+10 ± 20	<0.001
Frequency reduction	−2.2 ± 2.0	−1.1 ± 1.5	−0.3 ± 1.0	<0.001
Urgency reduction	−2.5 ± 2.3	−1.2 ± 1.8	−0.4 ± 1.2	<0.001
Incontinence reduction	−2.0 ± 2.1	−1.0 ± 1.6	−0.2 ± 1.1	<0.001
QoL improvement	+10.2 ± 11.5	+5.8 ± 8.2	+1.5 ± 5.0	<0.001
Compliance change	+4.5 ± 3.8	+2.2 ± 3.0	+0.8 ± 2.1	<0.001
Detrusor reduction	−6.5 ± 6.8	−3.2 ± 5.5	−1.1 ± 3.8	0.002
Nocturia change	−0.9 ± 1.2	−0.4 ± 0.9	−0.1 ± 0.5	0.004
Voided volume change	+55 ± 50	+28 ± 32	+12 ± 20	<0.001
Flow rate change	+2.5 ± 3.0	+1.2 ± 2.4	+0.3 ± 1.5	0.006
Hesitancy improvement (%)	25.2	16.5	8.2	0.032
Pain reduction (%)	20.5	12.4	5.8	0.041
Sensitivity reduction	−1.8	−0.9	−0.3	0.003
OAB reduction	−2.0	−1.1	−0.4	0.002
Pad reduction	−1.0	−0.5	−0.1	<0.001
Sleep improvement	+1.5	+0.7	+0.2	0.005
Anxiety reduction (%)	10.5	5.2	2.1	0.048
Depression reduction (%)	9.2	4.8	1.9	0.052
Adherence (%)	82.5	68.4	52.1	<0.001
High diet (%)	55.2	32.5	18.4	<0.001
Moderate fluid (%)	60.5	48.2	35.1	0.036
Low fluid (%)	15.2	28.5	42.8	0.039
High fiber (%)	62.8	45.1	28.2	0.034
High caffeine (%)	18.5	28.2	40.5	0.038
Clinical success (%)	100	—	—	<0.001

### 3.12 Male-only voiding-phase

The male subjects diagnosed with BPH had significantly poor voiding phase function than those without BPH in terms of maximum flow rate (18.0 ± 5.0 vs. 20.5 ± 5.8 mL/s), residual urine (51 ± 23 vs. 42 ± 19 mL), and voiding time (33 ± 11 vs. 28 ± 9 s). Symptoms like hesitation, intermittency, and straining were also significantly increased in the BPH population (all *p* < 0.05).

There was consistency in the finding that high dietary intake resulted in good outcomes in the urodynamic study of patients regardless of their stratification. Patients under high dietary intake had high flow rates, low residual urine, increased bladder compliance, and low storage phase detrusor pressure than the subjects from the low dietary intake category. Besides, high diet correlated with lower OAB, nocturia, and QoL scores (*p* < 0.05).

Statistically, there were significant differences among patients in the dietary intake strata in terms of the improvements observed in the bladder function test regardless of BPH-induced poor voiding function (Table 12).

**Table 12:** Male-only voiding-phase parameters by BPH status and dietary quality.

Parameter	Total (male)	Low diet	Moderate diet	High diet	*p*-value
Age (years)	56.9 ± 11.7	58.1 ± 12.0	56.2 ± 11.5	55.8 ± 11.2	0.041
BMI (kg/m^2^)	27.5 ± 4.7	28.0 ± 4.9	27.3 ± 4.6	26.8 ± 4.4	0.036
Symptom duration (years)	4.0 ± 2.5	4.3 ± 2.7	3.9 ± 2.4	3.6 ± 2.2	0.048
Frequency/day	10.1 ± 2.6	10.7 ± 2.8	9.4 ± 2.4	9.8 ± 2.5	0.031
Nocturia/night	2.3 ± 1.2	2.5 ± 1.3	2.1 ± 1.1	2.0 ± 1.0	0.028
Hesitancy (%)	23.9	26.8	21.5	19.2	0.019
Straining (%)	20.1	22.9	18.6	16.8	0.022
Intermittency (%)	24.8	27.5	22.0	20.5	0.017
Residual urine (mL)	47 ± 22	51 ± 23	45 ± 21	42 ± 19	0.015
Voided volume (mL)	225 ± 65	212 ± 60	236 ± 68	241 ± 70	0.021
Max flow rate (mL/s)	19.0 ± 5.3	18.0 ± 5.0	20.0 ± 5.6	20.5 ± 5.8	0.011
Voiding time (sec)	30 ± 10	33 ± 11	29 ± 9	28 ± 9	0.018
Storage detrusor pressure	43.0 ± 12.1	45.6 ± 13.0	42.0 ± 11.6	41.5 ± 11.4	0.029
Bladder compliance	20.1 ± 6.4	18.8 ± 6.1	20.3 ± 6.6	21.0 ± 6.8	0.033
OAB score	6.8 ± 2.1	7.4 ± 2.3	6.6 ± 2.0	6.2 ± 1.9	0.027
QoL score	59.2 ± 12.0	56.0 ± 12.8	59.0 ± 11.7	61.5 ± 11.3	0.024
Pad use/day	1.9 ± 1.2	2.2 ± 1.4	1.9 ± 1.2	1.7 ± 1.1	0.030
Sleep disturbance (%)	38.5	42.0	37.2	34.5	0.034
Anxiety (%)	26.2	28.8	25.4	23.6	0.062
Clinical success (%)	61.0	52.0	64.5	66.8	0.018

## 4 Discussion

The current research demonstrates that dietary quality, together with fluid intake, is an essential factor in determining urodynamic outcomes and functional improvements in patients undergoing bladder rehabilitation. The results of the study confirm previous research findings by showing how diet and hydration patterns affect both clinical outcomes and specific symptoms.

The initial measurements demonstrated significant differences in both nutritional intake and fluid consumption among the study participants. Participants with better dietary habits consumed more fiber, protein, potassium, and whole-grain products, which previous research has established as necessary nutrients for improved urinary tract function (14–16). The study established a strong association between fiber intake and increased bladder capacity and supported prior research demonstrating that dietary fiber helps alleviate constipation-related bladder problems (17, 18). The study found that higher potassium intake increased bladder capacity, as potassium helps regulate smooth muscle and detrusor function, as shown in previous urodynamic studies (19–21). Previous research has demonstrated that excessive sodium consumption decreases detrusor compliance and increases urgency, supporting the finding that high-salt diets worsen bladder over activity and nocturia symptoms (22–24).

Researchers determined that fluid intake is an essential factor that people can adjust to influence their initial and post-rehabilitation urodynamic results. People who drank moderate amounts of fluids showed better bladder capacity because they experienced fewer bladder-emptying episodes and fewer emergency bathroom needs. The research results confirm the findings of Wyman et al. (25), who showed that proper fluid intake management helps achieve optimal bladder filling, resulting in reduced nighttime bathroom needs. The study found that high fluid intake increased bladder capacity but did not yield consistent results, as people exhibited different bathroom usage patterns when drinking too much water, a pattern that study results confirmed through bladder training research (26, 27).

The post-rehabilitation assessment showed that all participants experienced substantial improvement in their functional abilities, with greater capacity, higher voided volume, and improved compliance with their dietary and fluid intake requirements. The research established that high diet quality with moderate-to-high fluid intake produced the greatest functional improvement because both dietary and hydration factors worked together to achieve this effect. The interaction between diet and hydration yielded results similar to previous research, including Croagh et al. (28), who found that combining dietary and fluid changes led to better symptom management than either method alone. The research results show that adherence acts as an essential mediator; multivariate regression analysis demonstrated that both diet and fluid adherence independently predicted functional improvement (*β* = 0.25 and 0.23, respectively; *p* < 0.001). The research results support the growing consensus among experts that patient behavioral adherence plays a crucial role in determining success in bladder rehabilitation (29).

The symptom-specific outcomes demonstrate the clinical value of these results. The study found that patients experienced substantial decreases in urgency, incontinence, nocturia, and pad use, resulting in improved physiological outcomes and patient-centered outcomes, including quality of life and sleep quality. Earlier research established that these different treatment methods must be integrated to achieve lasting results in both overactive bladder treatment and bladder rehabilitation programs, according to Hardacker et al. (30). The study results show that people experience less urgent bathroom needs after consuming caffeine and drinking liquids, because their eating habits and drinking times affect how their bodies perceive bladder sensations, according to research showing that bladder nerve pathways respond to different osmotic and chemical stimuli (31, 32).

The positive relationship between high protein consumption and improved outcomes in urodynamics, including higher maximum urinary flows, increased volume voided, and greater recovery during rehabilitation, can be attributed to the necessity of having enough protein intake to maintain muscle strength and integrity. Smooth muscles and pelvic floor muscles are required to efficiently contract the bladder and allow urine discharge. Muscle integrity will improve bladder evacuation and enhance the urinary tract function.

Participants consuming high amounts of proteins had better dietary habits and followed rehabilitation recommendations more strictly. High-protein diet is related to efficient metabolic regulation, low inflammation, and faster tissue healing, which can help recover bladder function during rehabilitation.

In addition, the current study found that the patients with higher dietary quality, especially with higher intake of proteins, reported lesser symptom burden, urgency and incontinence events, and achieved better quality of life. Such results indicate that protein intake may be contributing to urodynamic improvement through an indirect effect that is linked to other nutritional and behavioral aspects such as healthy diet, physical activity, and better fluid management techniques.

Secondly, sufficient protein intake is important for overcoming bladder impairment caused by obesity and diabetes. Obesity and diabetes were noted to have a negative influence on functional recovery in the multivariate analysis. Thus, the positive effect revealed by the current study should be regarded as an overall result of proper nutrition improvement of patients’ physiology and metabolism.

The current results highlight the significance of BPH as a key factor determining the presence of voiding phase dysfunction in male patients with LUTS due to impaired urinary flow rate, increased post-void residual urine volume, and greater levels of obstructive voiding symptoms. The described phenomena can be expected given the well-known pathogenesis of BPH bladder outlet obstruction.

At the same time, the results obtained within the current study also suggest that dietary quality may modify the storage and voiding phase urodynamics in men with LUTS. Improved bladder activity (flow rate increase, compliance improvement, and decreased symptom load) associated with higher dietary quality in this case can be regarded as the evidence of possible protective effects of nutrition in managing LUTS.

Several explanations for the demonstrated relationships can be offered. Firstly, there is evidence indicating that improved dietary habits contribute to the reduction of systemic inflammation and improve metabolism and neuromuscular activity in the lower urinary tract. Besides, healthier diets may have an indirect positive effect on bladder activity through better body weight control and insulin sensitivity.

The current strengths of the research require examination of several important details. The study found that people who drank large amounts of liquid developed better bladder capacity. However, their bladder control problems increased because they drank too much at night, which required researchers to analyze their drinking habits. The study found that high-protein and antioxidant consumption improved flow rates and overall function. However, people who consumed high-fat foods experienced slightly higher rates of incontinence, confirming earlier findings on how dietary fats affect detrusor over activity (33, 34). The results demonstrate that patients require tailored nutritional advice rather than standard dietary instructions.

The regression analysis found that three lifestyle factors, which people can change when they want to exercise and maintain their body weight and stop smoking, serve as separate factors that determine their ability to perform tasks. The research conducted by Bigajski et al. (35) established that metabolic health and physical activity levels determine the effectiveness of bladder function and rehabilitation outcomes. The researchers found that both baseline symptom severity and QoL scores were important predictors, supporting their finding that the initial clinical burden affects how patients respond to standard treatments. The research establishes that a nutritious diet and moderate fluid intake are essential factors that determine both urodynamic results and clinical outcomes during bladder rehabilitation. The research found that diet and hydration work together with adherence and lifestyle choices to affect bladder function recovery, which shows that rehabilitation needs to be customized and integrated through various treatment methods.

## 5 Strengths, limitations, and prospects

### 5.1 Strengths

- The study achieved strong statistical power because it included 2,400 patients, which allowed researchers to find links between their dietary and fluid practices and their urodynamic test results.
- The researchers conducted a comprehensive assessment of both initial dietary consumption and urodynamic measurement results, which permitted the examination of multiple factors.
- The researchers used diet quality and fluid consumption data to study how lifestyle choices affect their study participants.
- The study combined urodynamic assessments with symptom evaluations, quality of life measurements, and treatment adherence information to create a more clinically relevant assessment.
- The researchers used multivariate regression to identify which factors predicted functional improvements for bladder rehabilitation according to their evidence-based recommendations.
- The study created practical dietary recommendations through its assessment of dietary elements, which included fiber and protein as well as potassium and sodium content.

### 5.2 Limitations

- Participants in the study tended to remember food consumption from the past, which led to inaccurate intake estimates through both underreporting and overreporting.
- The research results do not apply to all groups because the study used specific demographic and cultural conditions to conduct its research.
- The post-rehabilitation assessment may not capture long-term sustainability of functional improvements.
- The multivariate analysis considered multiple factors, but the study still faced residual confounding because participants changed their medications and had additional health conditions.
- The study results lack hydration and nutrient status verification through biochemical methods, which restricts scientists from understanding specific biological processes.

### 5.3 Future prospects

- Execute longitudinal studies to assess the long-term effects of functional improvements and the rates of their return to the original condition.
- Conduct interventional research studies that will test different dietary patterns and fluid consumption methods to determine their direct effects.
- The organization will create personalized nutrition and hydration programs that will work together with existing behavioral and medication methods.
- Researchers will monitor participants’ drinking behavior and food consumption patterns and their actual urodynamic measurements through telehealth systems and wearable technology.
- The study will use metabolic, inflammatory, and microbiome biomarkers to reveal the biological mechanisms through which diet affects bladder function.
- Research will include multiple ethnic groups and different age ranges to create findings that apply to everyone and benefit population health.

## 6 Conclusion

The research results demonstrate that dietary quality and fluid consumption are directly correlated with urodynamic results and the functional performance of patients undergoing bladder rehabilitation. The study found that improvements in bladder capacity, compliance, voiding efficiency, and symptom reduction were associated with better diet quality, higher fiber and potassium intake, and adequate hydration. In contrast, excessive nighttime fluid intake, high sodium intake, and poor treatment adherence were associated with worse treatment outcomes. The study found that combined diet–fluid interventions yielded greater functional improvements than single interventions, demonstrating that comprehensive lifestyle changes are essential for effective bladder-control treatment. The multivariate analysis showed that diet and hydration adherence function as separate predictive factors for clinical success, thereby establishing their essential role in the development of personalized rehabilitation programs. The study results demonstrate that bladder rehabilitation programs should implement structured dietary and hydration guidelines to support functional recovery, enhance quality of life, and reduce symptom distress in patients with lower urinary tract dysfunction.

## Data Availability

The raw data supporting the conclusions of this article will be made available by the authors, without undue reservation.
